# Establishment of a Veterinary Institution in Prague

**Published:** 1897-05

**Authors:** 


ESTABLISHMENT OF A VETERINARY INSTITUTION IN PRAGUE. At the session of the Bohemian Landtag of January 17th the following resolution of Representative Dworals, concerning the establishment of a veterinary institute, came up for first reading: That the Provincial government be authorized to enter into negotiations with the Imperial government, in order that a Bohemian veterinary institute may be founded in Prague, to fill the long-felt want in the kingdom of Bohemia of veterinary surgeons thoroughly trained in all the practical departments of the science. In speaking of the measure Dr. Dworals dwelt upon the necessity of prolonging the course to four years. The final aim of veterinary medicine is not merely to protect the live-stock which the nation possesses, but also to protect humanity from the baneful influence of epidemics among the domestic animals. For this, not empiricism, but the most exact science is necessary.—*Thierärztliches Centralblatt*, February 15, 1896.

